# Cardiovascular toxicity of tisagenlecleucel in children and adolescents: analysis of spontaneous reports submitted to FAERS

**DOI:** 10.3389/fimmu.2025.1499143

**Published:** 2025-01-22

**Authors:** Ganggang Wang, Lin Su, Yanjun Liu, Xiaohan Yang, Yi Li, Qi Mei, Wen Gao

**Affiliations:** ^1^ Department of Lymphatic Oncology, Cancer Center, Shanxi Bethune Hospital, Shanxi Academy of Medical Sciences, Third Hospital of Shanxi Medical University, Tongji Shanxi Hospital, Taiyuan, China; ^2^ Department of Respiratory and Critical Care, Fourth People’s Hospital of Jinan City, Jinan, Shandong, China; ^3^ Department of Cardiology, Fourth People’s Hospital of Jinan City, Jinan, Shandong, China; ^4^ Department of Oncology, Tongji Hospital, Tongji Medical College, Huazhong University of Science and Technology, Wuhan, Hubei, China; ^5^ Cancer Center, Shanxi Bethune Hospital, Shanxi Academy of Medical Sciences, Tongji Shanxi Hospital, Third Hospital of Shanxi Medical University, Taiyuan, Shanxi, China

**Keywords:** tisagenlecleucel, Adverse Event Reporting System, data mining, cardiovascular adverse event, child

## Abstract

**Background:**

The advent of tisagenlecleucel has been a major advance in the pharmacological treatment of relapsed/refractory B-cell acute lymphoblastic leukemia in children and adolescents. However, further research is required to better define its safety profile.

**Objectives:**

To determine the cardiovascular toxicity of tisagenlecleucel in children and adolescents.

**Methods:**

The US Food and Drug Administration’s Adverse Event Reporting System was searched to identify cardiovascular adverse events (CVAEs) related to tisagenlecleucel in pediatric patients up to the age of 18 years.

**Results:**

The median time to onset of tisagenlecleucel-associated CVAEs was shorter than that of tisagenlecleucel-associated non-CVAEs (3 days [interquartile range (IQR) 1, 6] vs. 7 days [IQR 2, 54]). The median time to onset was longer in patients with fatal CVAEs than in those with non-fatal CVAEs (4 days [IQR 1, 12.5] vs. 2 days [IQR 1, 4]). The most frequently reported CVAEs were mitral valve disease, hypotension, and capillary leak syndrome. Patients who developed shock had the highest mortality rate (66.67%). Concomitant use of medication for a neurological disorder was an independent risk factor for CVAEs, and concomitant use of medication for a respiratory disease was an independent risk factor for fatal CVAEs. Most CVAEs were associated with cytokine release syndrome, and older patients had a more favorable prognosis.

**Conclusions:**

Children and adolescents who receive tisagenlecleucel should be closely monitored for CVAEs, particularly during the first week of treatment.

## 1 Introduction

Tisagenlecleucel, previously known as CTL019, is an anti-CD19 chimeric antigen receptor (CAR) T-cell therapy that is the sole CAR-T product approved in young age for the management of relapsed/refractory B-cell acute lymphoblastic leukemia (B-ALL), with additional indications encompassing relapsed/refractory diffuse large B-cell lymphoma and relapsed/refractory follicular lymphoma in adults (1–3). However, it is worth noting that on January 19, 2024, the US Food and Drug Administration (FDA) mandated updated labeling of the CAR T-cell products already available in the marketplace to incorporate a black box warning about the risk of T-cell malignancy. This warning has heightened awareness of the need for careful consideration of the potential adverse events (AEs) of CAR T-cell therapy and their management.

CAR T-cells are administered as a single infusion after a short course of lymphodepleting chemotherapy, which is usually a combination of cyclophosphamide and fludarabine (4). This conditioning regimen determines different biological effects, such as lymphodepletion, eradication of immunosuppressive cells (regulatory T cells and myeloid-derived suppressor cells), modulation of tumor microenvironment and increased expansion and persistence of CAR-T cells (5–9). Cytokine release syndrome (CRS) and immune effector cell-associated neurotoxicity syndrome are common reversible CAR T-cell toxicities (10). Although CRS and immune effector cell-associated neurotoxicity syndrome have been extensively studied, clinical trials do not always include rare toxicities (11). Recent data suggest that the incidence of cardiovascular and pulmonary AEs with the commercially available CAR T-cell therapies is higher than 10% (12–17). Alvi et al. found that 28% of patients with severe CRS had a reduced ejection fraction (13), and Ganatra et al. identified new or worsening cardiomyopathy in 10% of recipients of CAR T-cell therapy (18). A combined analysis of the ELIANA (79 participants) and ENSIGN (58 participants) phase-2 clinical trials found that out of 137 children and young adults, 31% experienced cardiac issues, with 7% being grade 3 or 4. Arrhythmias were the most frequent, occurring in 29% of cases (19, 20). These reports of cardiac toxicity predominantly stem from the clinical trials involving CAR T-cell therapy in adult patients and from smaller single-center studies in pediatric populations. Therefore, a comprehensive understanding of the toxicity profile of CAR T-cell therapy in children and adolescents is important from a clinical perspective.

Post-marketing surveillance data from large repositories could help to identify AEs and inform clinicians of the risks of these events. In this study, we examined the cardiovascular toxicity of tisagenlecleucel in the largest sample size of children and adolescents to date by leveraging information in the FDA Adverse Event Reporting System (FAERS) database.

## 2 Methods

### 2.1 Study design and data source

The relationship between tisagenlecleucel and AEs affecting the cardiovascular system was assessed using a case/non-case study design with disproportionality analysis. The frequency of AEs specifically associated with the target drug (cases) was compared with that associated with other drugs (non-cases). A safety concern arose when the incidence of an AE was higher for the target drug than for other drugs. The data necessary for this analysis were sourced from the FAERS Quarterly Data Extract Files (available at https://fis.fda.gov/extensions/FPD-QDE-FAERS/FPD-QDE-FAERS.html). To ensure the inclusion of the most up-to-date reports, all documented cases in the FAERS database from the second quarter of 2017 to the fourth quarter of 2023 were extracted (21). This study did not require ethical approval or informed consent because it analyzed data from the FAERS database, which is publicly accessible and contains anonymized patient records.

### 2.2 Data extraction and descriptive analysis

The FAERS database acquires data from spontaneous reports, leading to potential duplication or withdrawal of reports. To address this problem, the FDA’s official guidance document outlines deduplication protocols and a roster of reports for removal. This study adhered closely to the FDA’s directives for data refining as stated on its official website. The refining procedures included eliminating duplicate entries using the approach endorsed by the FDA. Notably, we extracted the PRIMARYID, CASEID, and FDA_DT fields from the DEMO table and organized the reports on the basis of CASEID, FDA_DT, and PRIMARYID. When we encountered reports sharing the same CASEID, we retained the entry with the highest FDA_DT value. Similarly, for reports with identical CASEID and FDA_DT values, the entrywith the highest PRIMARYID value was preserved. Furthermore, from the initial quarter of 2019 onwards, each quarterly data set included a list of reports indicated for deletion.

Following deduplication, the reports were expunged in accordance with the CASEID listings found in the deletion report log. The deduplication and data refining processes were performed using the methods endorsed by the FDA (22).

### 2.3 Application of the medical dictionary for regulatory activities

The FAERS database uses the Medical Dictionary for Regulatory Activities (MedDRA) to encode AE titles. These titles are represented by preferred terms from the MedDRA within the FAERS database. The MedDRA is updated biannually, specifically in March and September, resulting in modifications to the classification of preferred terms and adjustments to the relevant organ systems. Therefore, we used the most recent edition of the MedDRA to cross-reference the preferred terms in the FAERS database. The preferred terms were reevaluated, and the corresponding system organ class and preferred terms from the latest MedDRA dictionary version were collected for subsequent examination. Specifically, preferred terms in “Cardiac disorders” and “Vascular disorders” within the system organ class were included.

### 2.4 Statistical analysis

Drug safety signals were detected using the reporting odds ratio (ROR). This method was used to compare the frequency of AEs associated with the target drug with that of AEs associated with all other drugs. A ratio that exceeded a predetermined threshold indicated an imbalance and potential generation of safety signals. The ROR method used positive signal detection criteria, which included having at least three reports and the lower limit of the 95% confidence interval (CI) for an ROR >1. Variables that demonstrated statistical significance in the univariate logistic regression analysis were subsequently incorporated into the multivariate logistic regression analysis. Univariate logistic regression analysis of age, sex, and drug combinations that included tisagenlecleucel and any of 14 different agents used to treat digestive, metabolic, and cardiovascular disorders was performed to identify risk factors for CVAEs. Variables with a p-value <0.05 were entered into multivariate logistic regression analysis. The univariate logistic regression analysis identified significant differences across 13 variables associated with the occurrence of CVAEs. These variables included dermatological medications, systemic corticosteroids (excluding sexual hormones and insulin), systemic anti-infective agents, musculoskeletal system medications, sensory organ system medications, antiparasitic, insecticidal, and anthelmintic drugs, digestive and metabolic system drugs, genitourinary system and sexual hormones, nervous system medications, cardiovascular system medications, respiratory system medications, and blood and hematopoietic organ medications. To evaluate the association different groups, we used Fisher’s Exact Test for small sample sizes or when expected frequencies were less than 5, and employed the Chi-square test for larger samples where the expected frequency in each cell was at least 5. Continuous data that were not normally distributed were compared between groups using the Wilcoxon two-sample test. The statistical analysis was performed using SAS version 9.4 (SAS Institute Inc., Cary, NC, USA), SPSS version 22.0 (IBM Corp., Armonk, NY, USA), and Microsoft Excel 2019 (Microsoft Corp., Redmond, WA, USA). A p-value <0.05 was considered statistically significant.

## 3 Results

### 3.1 Baseline characteristics of children and adolescents with tisagenlecleucel-associated cardiovascular AEs

Of the 10,139,894 patients with data recorded in the FAERS database during the study period, 386,547 were included in the analysis after exclusion of duplicate cases (1,760,590 reports).

Among the 568 patients under the age of 18 years who were identified to be using tisagenlecleucel, 187 (32.92%) experienced CVAEs and 381 (67.08%) experienced non-CVAEs. There were 116 male patients (62.03%) and 64 female patients (34.22%) in the group with CVAEs and 219 (57.48%) and 149 (39.11%), respectively, in the group with non-CVAEs. There was no statistically significant difference in sex distribution between these two groups (p>0.05). There was also no statistically significant difference in mean age between the group with CVAEs and the group with non-CVAEs (10 years vs 11 years; p>0.05; **Table 1**). The majority of reports originated from the USA (n=434, 76.41%), followed by Japan (n=22, 3.87%), Canada (n=20, 3.51%), Spain (n=17, 2.99%), and Australia (n=12, 2.11%). The remaining reports (11.09%) were from nine other countries. In total, 310 (54.58%) reports were from physicians, 137 (24.12%) were from pharmacists, and 4 (0.70%) were from an unidentified source.

**Table 1:** Demographic and clinical characteristics of patients with tisagenlecleucel-associated adverse events reported in the FAERS database.

Clinical characteristics	CVAEs (n=187)	Non-CVAEs (n=381)	Total (n=568)	P Value
**Gender, n (%)**				0.5280
Male	116(62.03%)	219 (57.48%)	335 (58.98%)	
Female	64 (34.22%)	149 (39.11%)	213 (37.50%)	
Missing	7 (3.74%)	13 (3.41%)	20 (3.52%)	
**Age (Year)**				0.3393
Median (Q1, Q3)	10 (6,14)	11 (6,14)	10.00(6.00,14.00)	
Country, n (%) (The top 5 are listed in descending order of the total number of reports)				
USA	156(83.42%)	278 (72.97%)	434 (76.41%)	
Japan	3 (1.60%)	19 (4.99%)	22 (3.87%)	
Canada	5 (2.67%)	15 (3.94%)	20 (3.52%)	
Spain	5 (2.67%)	12 (3.15%)	17 (2.99%)	
Australia	3 (1.60%)	9 (2.36%)	12 (2.11%)	
Received year, n (%)				
2017	0 (0.00%)	5 (1.31)	5 (0.88)	
2018	22 (11.76%)	27 (7.09%)	49 (8.63%)	
2019	23 (12.30%)	62 (16.27%)	85 (14.96%)	
2020	43 (22.99%)	117 (30.71%)	160 (28.17%)	
2021	32 (17.11%)	49 (12.86%)	81 (14.26%)	
2022	49 (26.20%)	74 (19.42%)	123 (21.65%)	
2023	18 (9.63%)	47 (12.34%)	65 (11.44%)	
**Reporter type, n (%)**				0.1665
Physician	84 (44.92%)	226 (59.32%)	310 (54.58%)	
Other health professional	21 (11.23%)	34 (8.92%)	55 (9.68%)	
Consumer	16 (8.56%)	46 (12.07%)	62 (10.92%)	
Pharmacist	64 (34.22%)	73 (19.16%)	137 (24.12%)	
Missing	2 (1.07%)	2 (0.52%)	4 (0.70%)	

CVAEs, cardiovascular adverse events. Non-CVAEs, non-cardiovascular adverse events.

Continuing our analysis, we focused on the 187 patients who experienced CVAEs. The AEs were fatal in 57 (30.48%) of these patients and non-fatal in 130 (69.52%). There were 39 male patients (68.42%) and 16 female patients (28.07%) in the group with fatal CVAEs and 77 (59.23%) and 48 (36.92%), respectively, in the group with non-fatal CVAEs. There was no statistically significant difference in sex distribution between the two groups (p>0.05). Furthermore, the mean age was significantly younger in patients with fatal CVAEs than in those with non-fatal CVAEs (7 years vs 11 years; p<0.05; **Table 2**).

**Table 2:** Demographic and clinical characteristics of patients with tisagenlecleucel-associated cardiovascular adverse events reported in the FAERS database.

Clinical characteristics	Fatal CVAEs (n=57)	Non-fatal CVAEs (n=130)	Total (n=187)	P Value
**Gender, n (%)**				0.4702
Male	39 (68.42%)	77 (59.23%)	116 (62.03%)	
Female	16 (28.07%)	48 (36.92%)	64 (34.22%)	
Missing	2 (3.51%)	5 (3.85%)	7 (3.74%)	
**Age (Year)**				0.0111
Median (Q1, Q3)	7.00 (4.00,14.00)	11.00 (8.00,14.00)	11.00 (6.00,14.00)	
Country, n (%) (The top 5 are listed in descending order of the total number of reports)				
USA	45 (78.95%)	111 (85.38%)	156 (83.42%)	
Spain	0	5 (3.85%)	5 (2.67%)	
Canada	4 (7.02%)	1 (0.77%)	5 (2.67%)	
UK	2 (3.51%)	2 (1.54%)	4 (2.14%)	
Australia	2 (3.51)	1 (0.77%)	3 (1.60%)	
Japan	1 (1.75%)	2 (1.54%)	3 (1.60%)	
Received year, n (%)				
2018	7 (12.28%)	15 (11.54%)	22 (11.76%)	
2019	8 (14.04%)	15 (11.54%)	23 (12.30%)	
2020	13 (22.81%)	30 (23.08%)	43 (22.99%)	
2021	6 (10.53%)	26 (20.00%)	32 (17.11%)	
2022	17 (19.82%)	32 (24.62%)	49 (26.20%)	
2023	6 (10.53%)	12 (9.23%)	18 (9.63%)	
**Reporter type, n (%)**				0.0007
Physician	19 (33.33%)	65 (34.76%)	84 (64.62%)	
Health professional	20 (35.09%)	38 (20.32%)	58 (44.62%)	
Other health professional	10 (17.54%)	11 (5.88%)	21 (16.15%)	
Consumer	6 (10.53%)	10 (5.35%)	16 (12.31%)	
Pharmacist	1 (1.75%)	5 (2.67%)	6 (4.62%)	
Missing	1 (1.75%)	1 (0.53%)	2 (1.54%)	

Fatal CVAEs, cardiovascular adverse events that caused death. Non-fatal CVAEs, cardiovascular adverse events that did not cause death.

The majority of reports originated from the USA (n=156, 83.42%), followed by Spain (n=5, 2.67%), Canada (n=5, 2.67%), the UK (n=4, 2.14%), Australia (n=3, 1.60%), and Japan (n=3, 1.60%). The remaining reports (5.88%) came from 19 other countries. Eighty-four reports (64.62%) were from physicians, 58 (44.62%) were from other health professionals, and 2 (1.54%) were from an unknown source.

### 3.2 ROR and number of reports of the preferred term for tisagenlecleucel-associated cardiovascular AEs

**Figure 1:**
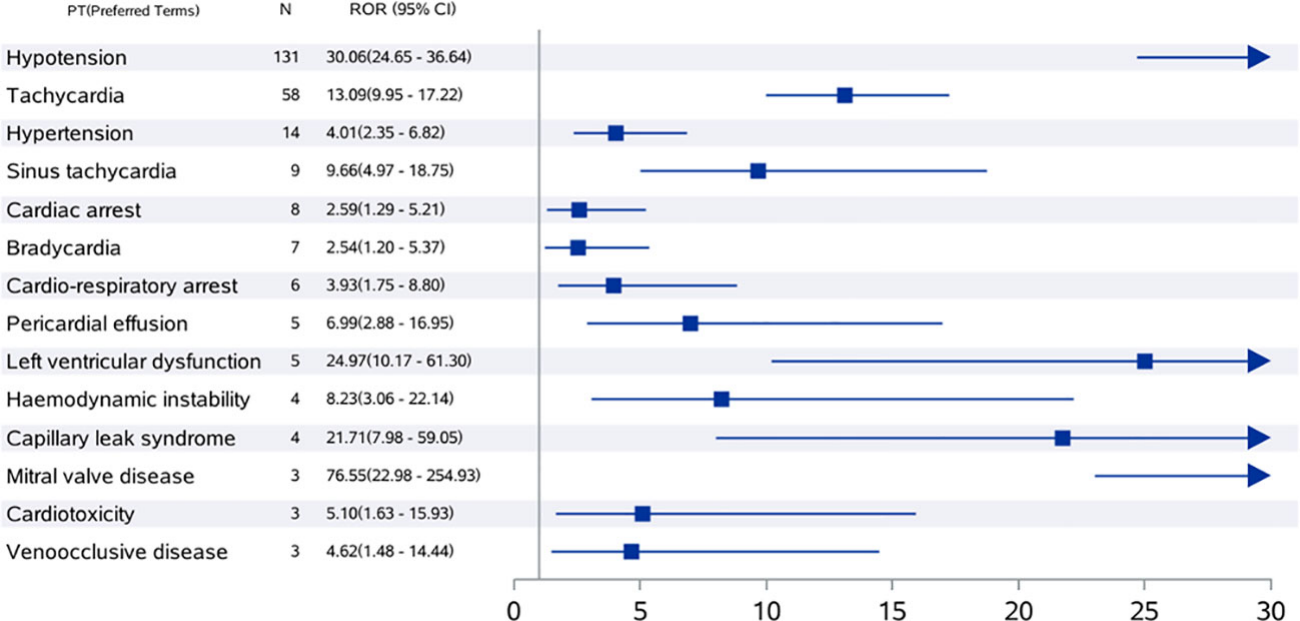
ROR values and number of reports of high-risk signal PT for tisagenlecleucel-associated cardiovascular adverse events in children and adolescents. The value displayed exceeds the highest value on the chart. CI, confidence interval; ROR, reporting odds ratio; PT, preferred term.

The numbers of reported cases and RORs for tisagenlecleucel in the target population were assessed using preferred terms (**Figure 1**). Hypotension was the most common CVAE with the highest ROR (n=30.06, 95% CI 24.65–36.64) and was followed by tachycardia, which had an ROR of 58 (n=58, 95% CI 9.95–17.22), and hypertension, which had an ROR of 4.01 (n=14, 95% CI 2.35–6.82). We also observed cardiorespiratory arrest and cardiac arrest.

### 3.3 Time to onset of tisagenlecleucel therapy-associated cardiovascular AEs

**Figure 2:**
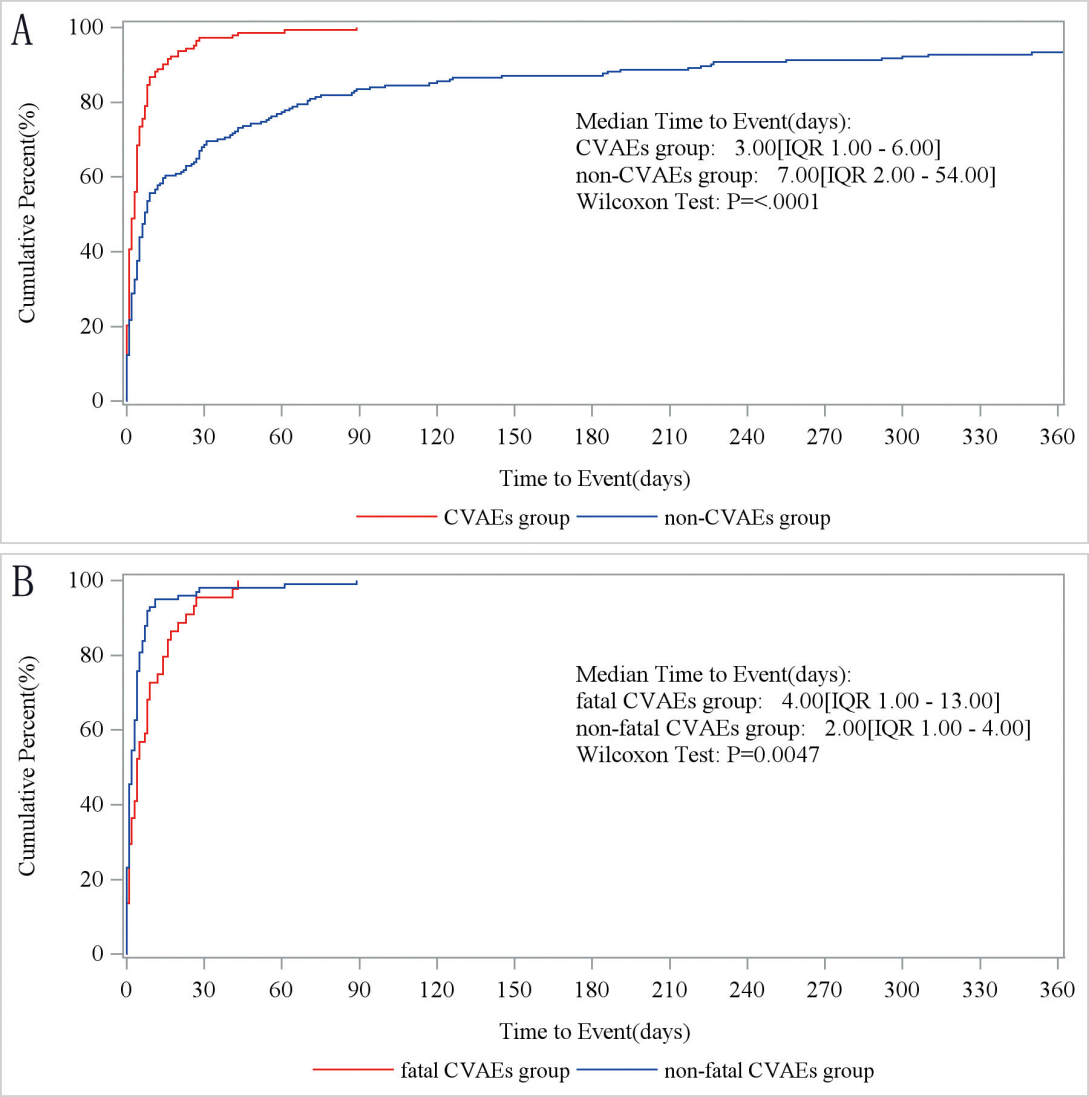
Time to onset of tisagenlecleucel-associated cardiovascular adverse events. **(A)** Times to onset of cardiovascular and non-cardiovascular AEs. CVAEs group, patients with cardiovascular AEs. Non-CVAEs group, patients with AEs involving other organ systems. **(B)** Times to onset of fatal and non-fatal cardiovascular AEs. Fatal CVAEs group, patients who developed cardiovascular AEs and died. Non-fatal CVAEs group, patients who developed cardiovascular AEs and survived. AE, adverse event.

Next, we compared the timing of occurrence between cardiovascular and non-cardiovascular AEs. We found that CVAEs manifested sooner after treatment with tisagenlecleucel than did non-CVAEs (3 days [IQR 2, 54] vs. 7 days [IQR 1, 6]; p<0.001; **Figure 2A**). Non-fatal CVAEs occurred later than fatal CVAEs after the start of treatment with tisagenlecleucel (4 days [IQR 1, 13] vs. 2 days [IQR 1, 4]; p<0.0047; **Figure 2B**).

### 3.4 Rates of overlap between CRS and tisagenlecleucel-associated cardiovascular AEs

**Figure 3:**
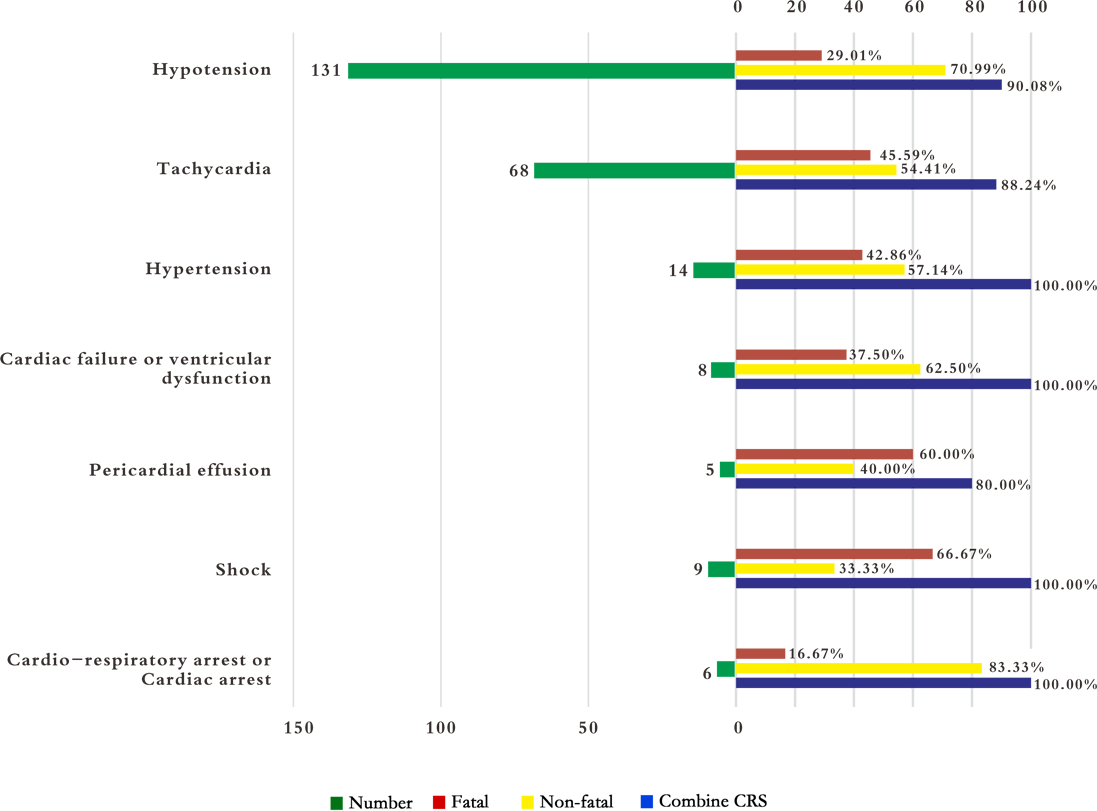
Rates of overlap between cytokine release syndrome and tisagenlecleucel-associated cardiovascular adverse events reported in children and adolescents. The number indicates the number of reported cases for PT from FAERS. Combined CRS, number of reported overlaps between CRS and PT from FAERS. CRS, cytokine release syndrome; FAERS, FDA Adverse Event Reporting System; PT, preferred term.

We observed a significant overlap between CVAEs and CRS (**Figure 3**). Notably, all manifestations of tisagenlecleucel-induced CVAEs, including hypertension, heart failure, tachycardia, pericardial effusion, shock, cardiopulmonary arrest, and cardiac arrest, were consistently accompanied by CRS. The mortality rate was lowest for cardiopulmonary or cardiac arrest (n=1, 16.67%) and highest in cases of shock (n=6, 66.67%). CRS also occurred in 90.08% of patients with hypotension and had a mortality rate of 29.01%. The mortality rate in patients with tachycardia was 45.59%.

### 3.5 Risk factors for tisagenlecleucel-associated cardiovascular AEs

Multivariate logistic regression analysis showed that patients receiving medication for neurological diseases were more prone to CVAEs (**Table 3**). To further analyze the factors related to fatal CVAEs induced by tisagenlecleucel, univariate logistic regression analysis was conducted using the method described above but with the exclusion of concurrent cardiovascular medicines (**Table 4**). This analysis revealed significant differences in age, dermatological medications, systemic corticosteroids (excluding sexual hormones and insulin), antineoplastic and immunomodulating agents, antiparasitic, insecticidal and anthelmintic drugs, genitourinary system and sexual hormones, digestive and metabolic system drugs, respiratory system medications, and blood and hematopoietic organ medications. Multivariate logistic regression showed that older age was associated with a lower risk of death and that patients taking tisagenlecleucel combined with medications for respiratory disease appeared to have a higher likelihood of death.

**Table 3:** Univariate and multivariate analysis of CVAEs induced by tisagenlecleucel.

Variable	Univariate		Multivariate				
	Waldχ2	P value	β	S.E	Waldχ2	P value	OR (95% CI)
Age	0.88	0.3802	–	–	–	–	–
Gender	1.24	0.2658	–	–	–	–	–
Dermatological medications	14.17	**0.0002**	-0.2204	0.5958	0.1369	0.7114	0.80 (0.25 - 2.58)
Systemic corticosteroids, excluding sexual hormones and insulin	5.68	**0.0172**	0.0958	0.4700	0.0415	0.8385	1.10 (0.44 - 2.76)
Systemic anti-infective agents	17.07	**<.0001**	-0.7346	0.4844	2.3005	0.1293	0.48 (0.19 - 1.24)
Antineoplastic and immunomodulating agents	1.75	0.1858	–	–	–	–	–
Musculoskeletal system medications	14.29	**0.0002**	0.2838	0.4771	0.3539	0.5519	1.33 (0.52 - 3.38)
Sensory organ system medications	18.59	**<.0001**	0.3174	0.7312	0.1884	0.6642	1.37 (0.33 - 5.76)
Antiparasitic, insecticidal, and anthelmintic drugs	10.99	**0.0009**	0.3423	0.5056	0.4584	0.4984	1.41 (0.52 - 3.79)
Digestive and metabolic system drugs	19.02	**<.0001**	0.4920	0.5735	0.7360	0.3909	1.64 (0.53 - 5.03)
Genitourinary system and sexual hormones	13.18	**0.0003**	-0.0386	0.4473	0.0075	0.9312	0.96 (0.40 - 2.31)
Nervous system medication	47.90	**<.0001**	1.5285	0.2325	43.2171	<.0001	4.61 (2.92 - 7.27)
Cardiovascular system medications	17.20	**<.0001**	0.4087	0.5064	0.6514	0.4196	1.50 (0.56 - 4.06)
Respiratory system medications	10.89	**0.0010**	-1.4175	0.6530	4.7128	0.0599	0.24 (0.07 - 0.87)
Blood and hematopoietic organs medication	19.45	**<.0001**	0.1779	0.5679	0.0982	0.7540	1.19 (0.39 - 3.64)
Other miscellaneous drugs	14.79	**0.0001**	0.2184	0.5542	0.1553	0.6935	1.24 (0.42 - 3.69)

CI, confidence interval; OR, odds ratio; S.E, standard error.

Bold texts indicates P < 0.05, signifying statistical significance.

**Table 4:** Univariate and multivariate analysis of fatal CVAEs induced by tisagenlecleucel.

Variable	Univariate		Multivariate				
	Waldχ2	P value	β	S.E	Waldχ2	P value	OR (95% CI)
Age	-2.87(t-test)	**0.0045**	-0.0909	0.0358	6.4554	**0.0111**	0.91 (0.85 - 0.98)
Gender	1.44	0.2294	–	–	–	–	–
Dermatological medications	5.75	**0.0165**	0.4581	0.8160	0.3152	0.5745	1.58 (0.32 - 7.83)
Systemic corticosteroids, excluding sexual hormones and insulin	10.55	**0.0012**	2.3239	1.0227	5.1635	0.0531	10.22 (1.38 - 75.82)
Systemic anti-infective agents	3.80	0.0514	–	–	–	–	–
Antineoplastic and immunomodulating agents	7.84	**0.0051**	0.0831	0.7951	0.0109	0.9168	1.09 (0.23 - 5.16)
Musculoskeletal system medications	3.06	0.0801	–	–	–	–	–
Sensory organ system medications	2.99	0.0836	–	–	–	–	–
Antiparasitic, insecticidal, and anthelmintic drugs	6.35	**0.0117**	-0.5554	0.8311	0.4466	0.5040	0.57 (0.11 - 2.93)
Genitourinary system and sexual hormones	4.10	**0.0428**	-1.7416	0.9377	3.4494	0.0633	0.18 (0.03 - 1.10)
Nervous system medication	1.92	0.1663	–	–	–	–	–
Digestive and metabolic system drugs	4.71	**0.0299**	0.9538	0.8298	1.3211	0.2504	2.60 (0.51 - 13.20)
Respiratory system medications	13.82	**0.0002**	0.5991	0.9946	7.3629	0.0032	1.82 (0.26 - 12.79)
Blood and hematopoietic organs medication	6.42	**0.0113**	-1.0995	1.1044	0.9911	0.3195	0.33 (0.04 - 2.90)
Other miscellaneous drugs	1.99	0.1580	–	–	–	–	–

CI, confidence interval; OR, odds ratio; S.E, standard error.

Bold texts indicates P < 0.05, signifying statistical significance.

## 4 Discussion

**Figure 4:**
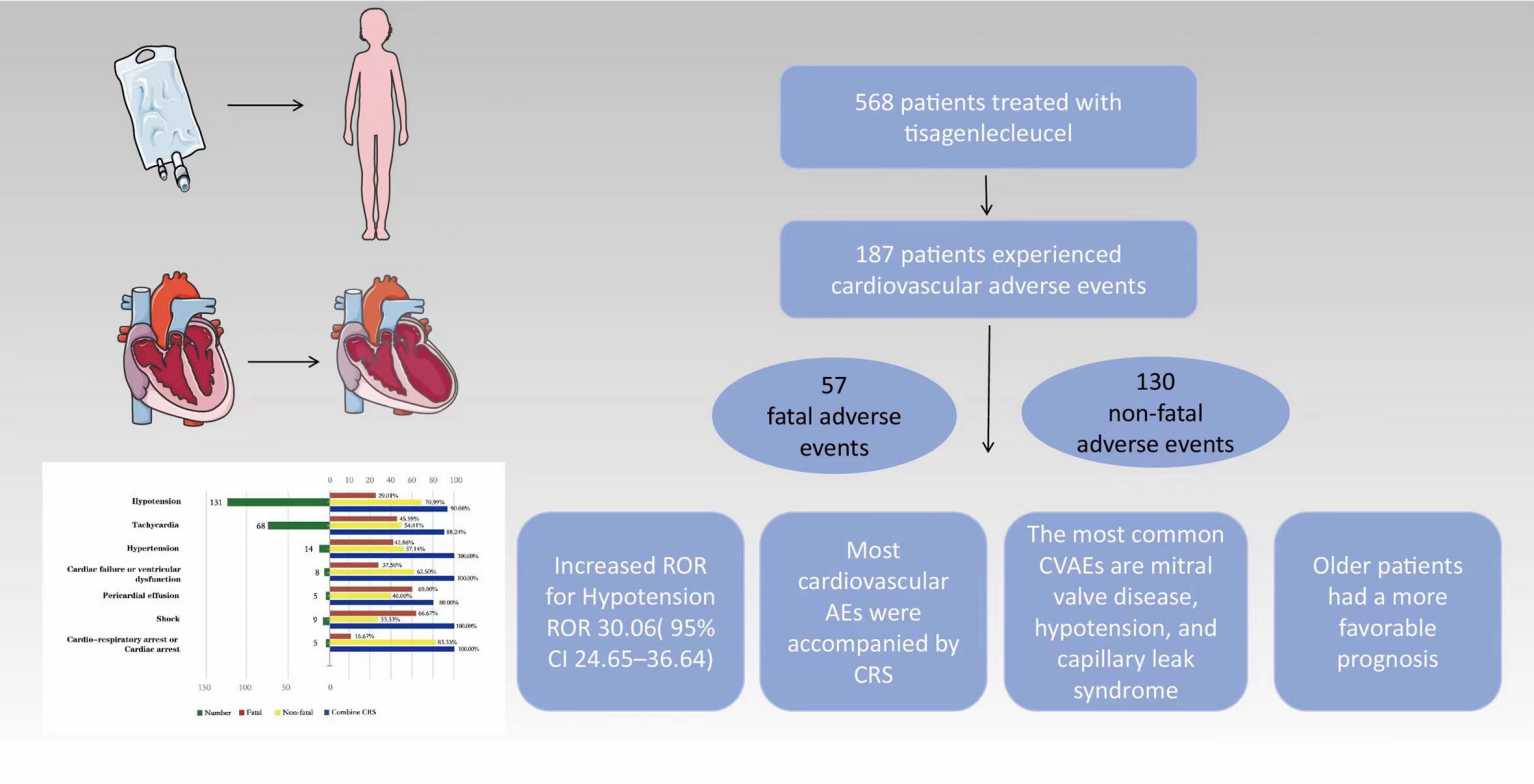
Cardiovascular adverse events and potential risk factors in children and adolescents with relapsed/refractory B-cell acute lymphoblastic leukemia treated with tisagenlecleucel based on the FAERS database. Cardiovascular adverse events (CVAEs) are observed in approximately one-third of patients. The most commonly reported CVAEs include mitral valve disease, hypotension, and capillary leak syndrome. The majority of CVAEs were associated with cytokine release syndrome, and older patients exhibited a more favorable prognosis.

To the best of our knowledge, this is the first post-marketing pharmacovigilance study to investigate tisagenlecleucel-related cardiovascular toxicity in children and adolescents. This study had several important findings (**Figure 4**). First, CVAEs associated with tisagenlecleucel were not uncommon in this age group, with 32.92% of reports to the FAERS mentioning such events. Second, most CVAEs associated with tisagenlecleucel occurred within 1 week of starting treatment and were accompanied by CRS. Third, patients using concomitant medications for disorders of the nervous system had a higher risk of CVAEs, while those who were receiving concomitant treatment for respiratory disease had a higher risk of fatal CVAEs. Fourth, there was no statistically significant difference in the frequency of CVAEs according to age group.

However, increasing age seemed to protect against fatal CVAEs. Tisagenlecleucel is the only CAR T-cell therapy approved for pediatric use. However, there are limited data on its cardiovascular safety in this population. Therefore, the findings of this study could have clinically important implications.

Tisagenlecleucel was the first CAR T-cell therapy approved for the treatment of relapsed/refractory B-ALL in children and young adults, later extended to include relapsed/refractory diffuse large B-cell lymphoma and follicular lymphoma in adults. This therapy has effectively addressed a treatment gap for this life-threatening condition predominantly affecting children and adolescents (23). A previous study of data from the FAERS database showed that the highest incidence of hypotension in patients receiving CAR T-cell therapy was in those aged 16 years or older (17). The present study identified hypotension to be the most prevalent cardiovascular AE in children treated with tisagenlecleucel, with 90.08% of patients also experiencing CRS. Tachycardia, closely linked with CRS, is another frequent CVAEs. Another study reported tachycardia to be a common symptom of CRS (24), with 88.24% of patients reporting tachycardia and CRS and all cases of tachycardia being associated with CRS. The Phase II ELIANA and ENSIGN trials evaluated tisagenlecleucel in 137 pediatric and young adult patients with relapsed/refractory B-ALL. Cardiotoxicities, primarily tachycardia (26%), were most common within the first 8 weeks. All cardiac events were transient and resolved, except for one case of persistent left ventricular dysfunction (19). These reports are consistent with our present finding that most CVAEs associated with tisagenlecleucel occur early after infusion of CAR T-cell therapy. There has been a report of two cases of acute heart failure in adults following CAR T-cell therapy (25). Both patients had lymphoma cells in their bone marrow at the time of CAR T-cell therapy and had previously received multiple lines of treatment. Furthermore, both patients experienced early-onset CRS, presenting with fever on the day of CAR T-cell infusion. Treatment with tocilizumab improved their heart function. A retrospective study found that six of 52 patients (median age, 13.4 years; range, 4.2–30.3 years) developed cardiac insufficiency accompanied by CRS, with four of these cases occurring 28 days post-CAR T-cell infusion (26). However, heart function returned to normal in these patients. A meta-analysis of 25 clinical studies found that CAR T-cell therapy in cancer patients was associated with an 8% incidence of cardiovascular dysfunction and a 5.3% incidence of heart failure. (27). These findings suggest that, in practical settings, CAR T-cell therapy is associated with an increased likelihood of heart failure. Eight of the patients in our study developed heart failure, which was complicated by CRS in all cases, resulting in a mortality rate of 37.5%.

A case report detailed a preschool-aged girl with B-ALL who developed severe septic shock after lymphocyte-depleting chemotherapy. Post tisagenlecleucel treatment, she experienced CRS with cardiac insufficiency and lymphadenopathy, leading to renovascular damage. Tocilizumab improved her condition, and she was discharged in good health. However, she later died on day 208 post-infusion due to cardiac arrest of unknown cause. (28). The meta-analysis mentioned earlier found an incidence of cardiac arrest of 1.3% following CAR T-cell therapy (27). In our study, six patients under 18 years old experienced cardiac or cardiorespiratory arrest after tisagenlecleucel treatment, all complicated by CRS, resulting in a mortality rate of 16.67%.

Research on hypertension induced by CAR T-cell therapy is limited. In a previous study, school-aged children who experienced cardiac arrest on tisagenlecleucel also had hypertension and were treated with amlodipine to manage their blood pressure (28). In our study, we identified five cases of hypertension accompanied by sinus tachycardia or tachycardia (four cases complicated by CRS) and five cases of hypertension with hypotension (all five cases complicated by CRS, three with neurological toxicity, and one with renal injury).

Hypertension may contribute to the development of CRS via release of stress hormones such as catecholamines (29). However, a β1-adrenergic receptor blocker was reported to mitigate the CRS response (30). These findings suggest an association between hypertension and CRS, although further investigation is required to establish the specific mechanism and a causal relationship. Close monitoring of blood pressure is recommended when administering tisagenlecleucel in children because hypertension may be more prevalent in clinical practice than has been reported.

A retrospective cohort study investigated 187 patients who underwent CAR T-cell therapy for refractory/relapsed aggressive non-Hodgkin lymphoma (14). The presence of cardiovascular risk factors at baseline and cardiovascular disease were found to increase the risk of developing CAR T-cell-related cardiomyopathy. Patients who developed cardiomyopathy required more supportive care after CAR T-cell infusion. While the left ventricular ejection fraction often recovered, cardiac dysfunction persisted in 50% of cases. Five of the cases in our study developed pericardial effusion on tisagenlecleucel, four developed CRS, four had tachycardia, and two had mitral regurgitation. The mortality associated with these complications was 60%. Interestingly, cardiomyopathy was not identified as a high-risk signal in this study, which is in contrast with findings in adult patients (17).

Given the significant overlap and potential causal link between CVAEs and CRS (90.87%), it is important to maintain a heightened level of vigilance for the emergence of CVAEs in patients with CRS. This is consistent with the findings of previous studies (31, 32).The exact pathophysiological mechanisms remain unclear. CRS has many features in common with systemic inflammatory response syndrome and the ensuing septic cardiomyopathy (33). Recognition of pathogen-associated molecular patterns triggers immune activation, which leads to release of pro-inflammatory cytokines, resulting in microvascular dysfunction coupled with oxidative and nitrosative stress, mitochondrial dysfunction, and alterations in calcium handling (34–36). The inflammatory cytokines secreted mediate this myocardial dysfunction, with interleukin-6 likely playing a key role. The adverse effects of interleukin-6 on myocardial integrity in the context of meningococcal sepsis have been confirmed (30). Heightened expression of von Willebrand factor has been noted in patients with severe CRS (37). These patients have increased levels of angiopoietin-2, which facilitates capillary leakage, alongside reduced angiopoietin-1, resulting in an increased ratio of angiopoietin-2 to angiopoietin-1 (38).

Next, we sought to identify the risk factors for CVAEs and found that patients who were receiving concomitant levetiracetam, which is used to treat epilepsy, had a higher likelihood of CVAEs. To our knowledge, there are few reports of cardiotoxicity caused by levetiracetam, although it has been reported that a high intake of this agent appears to cause bradycardia and hypotension, which may be reactive to atropine and intravenous infusion. Echocardiographic findings indicate that the mechanism of action of levetiracetam may involve interaction with muscarinic receptors at high concentrations (39). The results of our research suggest that these patients may have more severe CRS and be predisposed to other systemic disorders, including CVAEs. The findings of another study suggested that levetiracetam could potentially trigger CVAEs via specific mechanisms (40), although experimental confirmation is needed. That study also demonstrated that patients who were concurrently using medications for respiratory disease were more likely to experience fatal CVAEs. It can be inferred that patients who experience both cardiovascular and respiratory AEs have a poorer prognosis. This finding is clinically important and suggests that we should focus on children who are on medication for respiratory disease and intervene as early as possible to reduce the risk of fatal CVAEs. The increased mortality rate associated with cardiovascular adverse events in patients concurrently using nervous system or respiratory system medications may be related to the coexistence of multiple systemic diseases in these patients or to the inherent cardiovascular toxicity of these medications. This hypothesis requires further validation through prospective clinical trials. Interestingly, our results show that as the patient age increased, the likelihood of lethal consequences decreased, indicating that older age protects against fatal CVAEs in pediatric patients who receive tisagenlecleucel. This observation may be attributed to the correlation between age and resistance to the AEs induced by tisagenlecleucel.

## 5 Limitations

Our study has several limitations. First, the FAERS database may not have included all patients with tisagenlecleucel, leading to potential underreporting. Second, reports from non-medical professionals may have introduced diagnostic bias. Third, the number of patients with tisagenlecleucel aged younger than 18 years in the FAERS database was limited, necessitating more AE reports for comprehensive pharmacovigilance analysis. Fourth, the prevalence of cardiovascular comorbidities may have been underestimated because of the inference of comorbidities based on drugs with documented cardiovascular indications.

Finally, causal relationships cannot be definitively established in observational studies. Further validation using independent data sources, along with insight into potential mechanisms and prevention of tisagenlecleucel-related toxicity, are essential to confirm the causal nature of these signals.

## 6 Conclusion

The only CAR T-cell therapy currently approved for use in children and adolescents is tisagenlecleucel. However, there is limited research on tisagenlecleucel-induced cardiotoxicity in pediatric patients. This study provides important insights into clinical management of this condition. Enhanced monitoring for cardiotoxicity is essential, particularly within the first week of treatment, in younger individuals, those with concurrent CRS, and those receiving levetiracetam or medication for respiratory disease. In this study, the median time to occurrence of a fatal CVAE was longer than that for a non-fatal CVAE (4 days vs 2 days). This implies that timely detection of clinical CVAEs and proactive intervention could potentially decrease the mortality rate.

## Data Availability

Publicly available datasets were analyzed in this study. This data can be found here: https://www.fda.gov/drugs/drug-approvals-and-databases/fda-adverse-event-reporting-system-faers.
